# Endogenous Opioids and Their Role in Stem Cell Biology and Tissue Rescue

**DOI:** 10.3390/ijms23073819

**Published:** 2022-03-30

**Authors:** Giovannamaria Petrocelli, Luca Pampanella, Provvidenza M. Abruzzo, Carlo Ventura, Silvia Canaider, Federica Facchin

**Affiliations:** 1 Department of Experimental, Diagnostic and Specialty Medicine (DIMES), University of Bologna, Via Massarenti 9, 40138 Bologna, Italy; giovannam.petrocell2@unibo.it (G.P.); luca.pampanella@studio.unibo.it (L.P.); provvidenza.abruzzo2@unibo.it (P.M.A.); federica.facchin2@unibo.it (F.F.); 2 National Laboratory of Molecular Biology and Stem Cell Bioengineering of the National Institute of Biostructures and Biosystems (NIBB)–Eldor Lab, at the Innovation Accelerator, CNR, Via Piero Gobetti 101, 40129 Bologna, Italy

**Keywords:** endogenous opioid peptides, opioid receptors, stem cells, proliferation, stress response, stem cell differentiation

## Abstract

Opioids are considered the oldest drugs known by humans and have been used for sedation and pain relief for several centuries. Nowadays, endogenous opioid peptides are divided into four families: enkephalins, dynorphins, endorphins, and nociceptin/orphanin FQ. They exert their action through the opioid receptors (ORs), transmembrane proteins belonging to the super-family of G-protein-coupled receptors, and are expressed throughout the body; the receptors are the δ opioid receptor (DOR), μ opioid receptor (MOR), κ opioid receptor (KOR), and nociceptin/orphanin FQ receptor (NOP). Endogenous opioids are mainly studied in the central nervous system (CNS), but their role has been investigated in other organs, both in physiological and in pathological conditions. Here, we revise their role in stem cell (SC) biology, since these cells are a subject of great scientific interest due to their peculiar features and their involvement in cell-based therapies in regenerative medicine. In particular, we focus on endogenous opioids’ ability to modulate SC proliferation, stress response (to oxidative stress, starvation, or damage following ischemia–reperfusion), and differentiation towards different lineages, such as neurogenesis, vasculogenesis, and cardiogenesis.

## 1. Introduction

### 1.1. Endogenous Opioid Peptides

Opioids are considered the oldest drugs known by humans and have been used for pain relief and sedation for several centuries. They are a class of compounds related in structure to the natural plant alkaloids which are extracted from the resin of the poppy plant (*Papaver somniferum*) [1]. Among them, morphine is the most common, active compound, which exerts its action in the central and peripheral nervous systems (CNS and PNS, respectively) through binding to the opioid receptors (ORs) [2]. Studies aimed at investigating the mechanisms of action and the activities of morphine led to the identification of endogenous ligands for ORs [3]. In 1975, two classes of endogenous peptides, methionine-enkephalin (met-enkephalin) and leucine-enkephalin (leu-enkephalin), were discovered [4]. Afterwards, many other endogenous opioid ligands were identified, including dynorphin and beta-endorphin [5,6]. 

**Figure 1:**
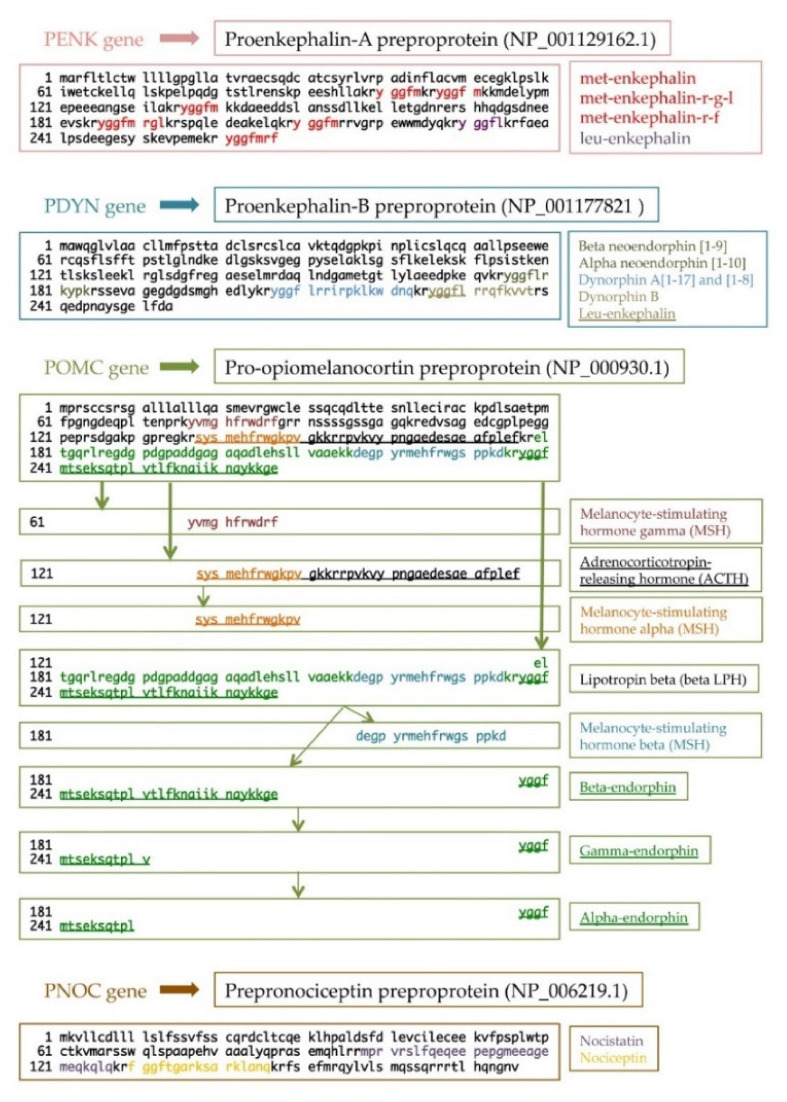
Schematic representation of human endogenous opioid families and their main functional peptides after precursor processing. For each family of peptides, the following information is reported: (i) the names of the genes (*PENK*, *PDYN*, *POMC*, and *PNOC*); (ii) the amino acid sequence of the preforms (NCBI Reference Sequence is reported in brackets next to the proform names); (iii) on the right, the names of the main functional peptides highlighted with a corresponding colour in the preform peptide sequence and in the isolated peptide sequence when it is required.

Nowadays, endogenous opioid peptides are divided into four families: enkephalins, dynorphins, endorphins, and nociceptin/orphanin FQ [7]. From a molecular point of view, each opioid peptide is synthesized as a prepro and a proform, creating functional peptides after precursor processing. All peptides share a common amino-terminal sequence, Tyr-Gly-Gly-Phe-(Met/Leu), namely, the opioid motif. For this reason, the same precursor may result in different opioid peptides (Figure 1) [8,9]. It is known that the Tyr and Phe amino acids of the opioid motif are essential for binding the opioid receptor, while the Gly pair acts as a spacer [10,11]. These peptides undergo post-translational, cell-specific modifications, such as acetylation, glycosylation, phosphorylation, and methylation. Such modifications determine changes in peptide efficacy and receptor affinity or selectivity and, consequently, changes in peptide physiological actions [12,13,14]. In the nervous system, endogenous opioid peptides play a crucial role in the modulation of emotional conditions, memory processes, neuroprotection, and analgesia. Moreover, these peptides are involved in the modulation of additional biological processes, such as embryonic development, angiogenesis, blood pressure regulation, respiratory control, feeding, peristalsis, pancreatic secretion, wound repair, and hepatoprotective mechanisms [11].

Enkephalins are encoded by the *proenkephalin* (*PENK*) gene, located on chromosome 8, and consist of three exons (gene ID: 5179). In humans and other mammals, the processing of the encoded precursor proenkephalin-A preproprotein (PENK, NP_001129162.1) generates six copies of met-enkephalin and one copy of leu-enkephalin [15] (Figure 1). Enkephalins are widely expressed in multiple brain regions and in the spinal cord, as well as in the adrenal medulla, endocrine tissues, and their target organs [16]. Enkephalins are degraded through the hydrolysis of the pentapeptide at the Tyr–Gly bond. Then, the molecules are reduced into shorter peptides (from two to four amino acids) by enkephalinases and aminopeptidases [10]. Since these small peptides have a short half-life both in vivo and in vitro, researchers also use synthetic forms of enkephalins, which are much more stable. Among them, the most common are DAMGO ([D-Ala^2^, N-MePhe^4^, Gly-ol]-enkephalin) [17,18], DPDPE ([D-Pen^2^, D-Pen^5^]-enkephalin) [19,20], and DADLE ([D-Ala^2^, D-Leu^5^]-enkephalin) [21,22].

Dynorphins, encoded by the four-exon *prodynorphin* (*PDYN*) gene located on chromosome 20 (gene ID: 5173), include bioactive peptides, such as dynorphin-A [1–17], dynorphin-A [1–8], dynorphin-B [1–13], alpha- and beta-neoendorphin, and leu-enkephalin [23,24,25]; all of them are generated by post-translational processing of the large precursor proenkephalin-B preproprotein (PDYN, NP_001177821) [26], which is processed by the prohormone convertases (PC), PC1 and PC2, and by carboxypeptidases [27,28] (Figure 1). Dynorphins are mainly expressed in the CNS, especially in the amygdala, entorhinal cortex, dentate gyrus, and nucleus accumbens, where they modulate processes related to learning and memory, emotional control, stress response, and pain [29]. Moreover, it was reported that myocardial cells express the *PDYN* gene [30], underlying the role of dynorphin-B in the control of the cardiovascular system and heart function [31].

Endorphins are encoded by the *pro-opiomelanocortin* (*POMC*) gene, located on chromosome 2, and consist of four exons (gene ID: 5443). Endorphins derive from the precursor pro-opiomelanocortin preproprotein (POMC, NP_000930.1), which is synthesized in the pituitary gland, as well as in keratinocytes [32] and the immune system [33]. POMC is processed by PCs into functionally active peptides, mainly melanocyte-stimulating hormone gamma (MSH), adrenocorticotropin-releasing hormone (ACTH), and beta-lipotropic hormone (beta-LPH). Endorphins derive from the proteolytic processing of beta-LPH. They are further divided into alpha-endorphins, beta-endorphins, and gamma-endorphins (Figure 1). The beta- and gamma-endorphins are the longest chains, and their sequences comprise the sequence of alpha-endorphins. Among the three endorphin types, beta-endorphins are the most studied: the beta-endorphin is one of the neuropeptides associated with states of pleasure, such as exercise-induced euphoria, love, sex, laughter, and appetite for food [34]. The pain relief experienced as a result of endorphin release was determined to be greater than that of morphine [35].

Nociceptin, or orphanin FQ (N/OFQ), is a small opioid peptide, discovered for the first time independently by two groups, hence, the double name [36,37]. The *prepronociceptin* (*PNOC*) gene consists of five exons and is located on chromosome 8 (gene ID: 5368). It encodes for the precursor prepronociceptin preproprotein (PNOC, NP_006219.1), which is then processed into active peptides by endopeptidase 24.11 [38,39] (Figure 1). N/OFQ is structurally similar to dynorphin-A but differs from the classical opioid peptides by the presence of a Phe instead of a Tyr at the amino terminus. This peculiar feature may explain why N/OFQ has no affinity for binding to the classical ORs [40,41]. N/OFQ is expressed in the human CNS and in immune cells. In the CNS, N/OFQ modulates several biological functions, such as pain transmission, stress and anxiety, learning and memory, locomotor activity, and food intake. Concerning the PNS, this peptide influences the activity of cardiac, digestive, secretory, renal, respiratory, and immune systems [42].

### 1.2. Opioid Receptors

Endogenous opioid peptides (and exogenous opioids) exert their action through the opioid receptors. ORs are transmembrane proteins belonging to the super-family of G-protein-coupled receptors (GPCRs), which are widely studied due to their key role in mood disorders, drug abuse/addiction, and pain management [43,44,45]. They are expressed not only in the CNS but also in many other districts, such as the gastrointestinal tract, the heart, and in the cells of the immune system [46,47,48]. There are four subtypes of OR: δ opioid receptor (DOR), μ opioid receptor (MOR), κ opioid receptor (KOR), and nociception/orphanin FQ (NOP) receptor. Although each receptor is encoded by a specific gene (*Oprd1, Oprm1, Oprk1,* and *Oprl1*, respectively), analysis of the amino acid composition revealed that they exhibit a more than 60% identical sequence identity [49,50,51] and share many similar molecular characteristics, providing the basis for novel drug design [52,53,54,55]. Once activated by agonists, all four ORs, coupled to the inhibitory G proteins, undergo a conformational change in their transmembrane domain, resulting in the activation of defined pathways. The associated G proteins inhibit the adenylyl cyclase, leading to a reduction of intracellular cyclic adenosine monophosphate (cAMP) levels and a consequent modulation of neural functions [56]. However, DOR, MOR, and KOR also have a constitutive activity, an agonist-independent mechanism that occurs during persistent pain and stress [57,58,59]. Initially, it was assumed that DOR and MOR were selectively activated by enkephalin and beta-endorphin, respectively, while KOR was activated by dynorphin [60]. However, it was demonstrated that DOR is also activated by beta-endorphin and dynorphins, which bind MOR with high affinity as well [14,61].

### 1.3. Stem Cells

Among all the cell types forming the body’s tissues, stem cells (SCs) are the subject of great scientific interest due to their peculiar features. In fact, they are characterized by two important properties: the ability to self-renew and the ability to differentiate into different cell types [62]. SCs can be divided into embryonic and adult SCs. The first are pluripotent cells, capable of differentiating into the mature cell types belonging to the three germ layers. Adult SCs are rare, quiescent cells with a more limited self-renewal and differentiation capacity [63,64]. In adult tissues, these cells provide physiological tissue homeostasis [65]. Although the mechanisms orchestrating the biology of SCs are not completely understood, it is suggested that their fate strongly depends on the interactions with their microenvironment, called the niche. Increasing evidence states that the niche, consisting of other non-SCs, the extracellular matrix, and signaling factors, in combination with the intrinsic characteristics of SCs, consistently defines their properties and potential [66,67]. Within this frame, SCs represent a particularly attractive tool for therapeutic applications and regenerative medicine. SC therapy showed interesting results in many diseases, such as chronic myeloid leukemia [68,69], cirrhosis [70,71], and heart failure [72,73]. Further applications in regenerative medicine include bone, cartilage, skin, and corneal regeneration [74]. Nevertheless, the use of SCs in regenerative medicine still has limits. In fact, the in vitro maintenance of SCs alters their differentiation and self-renewal ability due to the cellular senescence along culture passages [75,76]. Investigating molecules and strategies that can obviate these limits is currently an open field.

The aim of the present review is to provide a comprehensive view of the advances on the role of endogenous or synthetic opioid peptides in modulating different SC features. Owing to the multiplicity of the opioid types, of their receptors, and of the context of action, we decided to focus our discussion on two major areas: proliferation and response to stress (as oxidative stress, starvation, or damage following ischemia–reperfusion) on the one hand, and differentiation towards different lineages, such as neurogenesis, vasculogenesis, and cardiogenesis, on the other.

## 2. Endogenous Opioids Modulate Stem Cell Proliferation and Cell Stress Response

**Figure 2:**
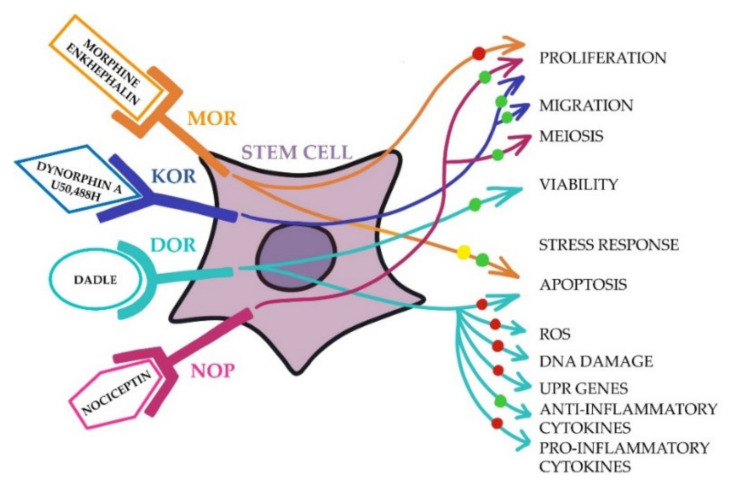
Schematic representation of the opioid receptors’ (MOR, KOR, DOR, and NOP) role in stem cell (SC) proliferation and stress response. Receptor agonists and cell responses written in the figure are obtained from the manuscript information. ”Stem cell” represents all types of SC (or SC-derived progenitors) described in the manuscript. All specifications written under “stress response” indicate the activities investigated in the context of the stress response. Red circle: inhibition; green circle: promotion; yellow circle: not conditioning; MOR: μ opioid receptors; KOR: κ opioid receptors; DOR: δ opioid receptors; NOP: nociception/orphanin FQ receptor; ROS: reactive oxygen species; UPR: unfolded protein response.

The opportunity to modulate SC proliferation and stress response represents one of the main goals of biological SC research aimed at improving the efficiency of SC transplantation. Here, we discuss the major outcomes of studies committed to evaluating the role of endogenous opioid peptides on these SC features according to the approach depicted in Figure 2 and summarized in Table 1.

In 2000, Hauser and coworkers [77] isolated, from postnatal mice, cerebellar external granular layer (EGL) neuronal precursors and demonstrated that these cells expressed proenkephalin-derived peptides, as well as specific receptors MOR and DOR, but an inappreciable amount of KOR. When these cells were treated with morphine (but not with met-enkephalin), a significant reduction in cell proliferation was observed, while the cell viability was not affected.

Two years later, Rozenfeld-Granot and colleagues [78] investigated the expression and the role of the MOR in hematopoietic stem cells (CD34^+^), in particular, in cord blood (CB) and peripheral blood (PB) CD34^+^ SCs isolated from the umbilical vein. Data obtained by microarrays, immunostaining, and fluorescence-activated cell sorting analysis showed that MOR was expressed in the cellular membrane of both cell types but three times more in CB-CD34^+^ cells than in PB-CD34^+^ cells. Therefore, to study whether the expression of the MOR into CB-CD34^+^ cells induces cell response via MAP kinase activation, as previously described in other cell models [79], cells were treated with met-enkephalin, a known MOR agonist. After 5 min of met-enkephalin stimulation, CB-CD34^+^ cells already showed an increase in the phosphorylated, activated form of mitogen-activated protein kinase (MAPK) and a moderate enhancement of p38 phosphorylation compared to the untreated cells [78]. Since MAPK cascades were shown to suppress apoptosis [80], researchers also investigated whether met-enkephalin may prevent apoptosis in irradiated CD34^+^ cells, but no significant differences were observed [78], suggesting that, in this cell type, met-enkephalin does not affect the apoptotic program.

In 2007, Sheng and collaborators [81] paid attention to neural stem cells (NSCs), well known to be involved in brain development and repair. They first showed a marked expression of KOR in highly enriched (90% nestin-positive) human fetal brain-derived NSCs. Then, they found that the KOR ligands, dynorphin-A [1–17] and trans-3,4-dichloro-N-methyl-N[2-(1-pyrolidinyl)cyclohexyl]benzeneacetamide methanesulfonate (U50,488), but not dynorphin-A[2–17], enhanced proliferation and migration of NSCs; these effects were partially abrogated when cells were treated with a KOR-selective antagonist, nor-binaltorphimine (nor-BNI), indicating, at least in part, the involvement of KOR in this mechanism.

On the other hand, Shoae-Hassani and colleagues, in 2011 [82], formulated an interesting hypothesis about a negative role of the opioid morphine on SC proliferation. They observed that morphine exposure reduced testosterone levels in the brain and spinal cord while enhancing the activity of enzymes, such as 5-alpha-redutase and aromatase, that convert testosterone into dihydrotestosterone (DHT) and estradiol, respectively [83,84,85]. Moreover, in NSCs, other evidence showed that some combinations of steroids, especially testosterone, were able to induce proliferation, while DHT had a negative effect on cell reproduction [86]. Finally, morphine induced an over-expression of the *p53* gene that could modulate SC apoptosis [87]. Combining all these data, the researchers hypothesized that, since morphine reduces testosterone levels, increases DHT levels, and over-expresses the *p53* gene, it might also prevent NSC proliferation [82].

Two years later, Willner and colleagues [88] also investigated the role of the opioid morphine on proliferation, apoptosis, and differentiation of the mouse CNS in the early prenatal period using the neural progenitor cells (NPCs) obtained from 14-day-old mouse embryos. NPCs were treated for 24 h with morphine sulfate at different concentrations. To evaluate single and associated parameters, treated NPCs were co-stained with specific markers of proliferation, apoptosis, or differentiation. These authors showed that, in the same cells, morphine decreased proliferation of NPCs and induced the apoptotic enzyme caspase-3 activity in a dose-dependent manner. Moreover, morphine increased the levels of active caspase-3 in proliferating cells, as evidenced by the increased number of double-positive cells in the morphine-treated groups. To uncover the receptor through which morphine acted as an anti-proliferative and pro-apoptotic peptide, cells were treated with the MOR antagonist naloxone; it abrogated the anti-proliferative and pro-apoptotic effects of morphine. Naloxone is a non-specific OR antagonist, but, since the main receptor expressed during prenatal mouse brain development is the MOR, the authors speculated that the primary action of the opioid system in the CNS during the early prenatal period is a MOR-mediated mechanism. They also demonstrated that morphine may induce differentiation of NPCs incorporating BrdU, as suggested by the significant increase in the percentage of BrdU-positive NPCs expressing the neuron-specific class III b-tubuline (Tuj1). To identify which cells are most sensitive to the apoptotic effects of morphine, authors proved that, in NPCs and astrocytes but not in Tuj1-expressing neurons, morphine causes increased caspase-3 activity [88].

The role of morphine on NSCs was also investigated in relation to cell distribution of insulin factor, insulin receptor, and insulin-like growth factors (IGF-1 and IGF-2) [89]. Researchers exposed NSCs isolated from rats to morphine for three days and observed an increase in cell apoptosis and a decrease in cell growth. They also observed a reduction in insulin biosynthesis, IGF-1 and IGF-2, and insulin receptor. When cells were exposed to naloxone, it restored the effects seen with morphine treatment [89].

In 2015, a role for nociceptin in self-renewal and differentiation of spermatogonial stem cells (SSCs) was reported [90]. SSC proliferation and differentiation are strictly controlled by Sertoli cells in the seminiferous tubules through the secretion of specific factors. Nociceptin was found among the most important, upstream, Sertoli-cell-related factors that, after being activated by follicle-stimulating hormone (FSH), regulate SSC self-renewal and spermatocyte meiosis [90]. As a matter of fact, nociceptin is expressed and secreted by Sertoli cells and, via NOP, induces spermatocytes to phosphorylate REC8, which activates the meiotic chromosome dynamics, thus, promoting the meiotic process [91,92].

The following reported studies indagated proliferation and cell stress response in human umbilical cord blood-derived mesenchymal stem cells (hUCB-MSCs) treated with a synthetic enkephalin, the peptide DADLE, in three different stress conditions.

In 2017, researchers [93] used hUCB-MSCs growing under serum starvation and treated them with DADLE, which is a specific DOR ligand, to investigate whether it displayed cytoprotective and anti-inflammatory effects. Serum starvation is commonly used to study cellular stress response, protein metabolism, apoptotic conditions, and cell death, and it can be used as an in vitro ischemic model [94,95]. Moreover, nutritional deprivation and inflammation-rich zones are the major obstacles to successful MSC transplantation. Thus, the improvement of MSC survival rate under such conditions is critical for ameliorating MSC-based tissue regeneration methods [96,97]. DADLE significantly increased cell viability and cell survival under serum-deprived stress conditions. The molecular mechanism by which DADLE exerts its action involved the activation of the pro-survival phosphoinositide 3-kinase/protein kinase B (PI3K/Akt) signaling pathway by the upregulation of PI3K subunit p110γ and the activation of Akt and the inhibition of the apoptotic cascade system by an increase in the anti-apoptotic B-cell lymphoma 2 (Bcl-2) and a decrease in both the pro-apoptotic Bcl-2-associated X protein/Bcl-2-associated death promoter (Bax/Bad) and the cleaved caspase-3 form.

Moreover, hUCB-MSCs treated with DADLE in serum-deprived conditions were induced to secrete increased levels of anti-inflammatory cytokines (interleukin 4, IL-4; interleukin 10, IL-10; and transforming growth factor-beta, TGF-β), and, when lipopolysaccharide-stimulated macrophages grew in this hMSC media, they released a reduced number of pro-inflammatory cytokines (interleukin 1, IL-1; tumor necrosis factor-alpha, TNF-*α*; interleukin 6, IL-6).

Finally, the cytoprotective and the anti-inflammatory effects of DADLE were both inhibited by the DOR-specific antagonist naltrindole but not by MOR and KOR agonists, indicating that these effects are mediated by DOR [93].

The same cellular model, hUCB-MSCs, and the same peptide, DADLE, were employed in another study performed in 2017 [98]. Cells were treated with H_2_O_2_, a reactive oxygen species (ROS)-inducing agent that has been established to induce oxidative stress conditions [99]. Researchers showed that DADLE improved the survival of hUCB-MSCs treated with H_2_O_2_ via the DOR receptor, as inferred by using specific receptor inhibitors. Moreover, hUCB-MSCs that were pre-treated with DADLE showed a significant reduction in intracellular ROS production, as well as the upregulation of anti-apoptotic marker Bcl-2 and a downregulation of pro-apoptotic proteins Bax and Bad. Finally, DADLE protected cells from H_2_O_2_-induced DNA damage, as was evidenced by a significant reduction in AP (apurinic/apyrimidinic) sites and the downregulation in the expression of key unfolded protein response (UPR) genes (*IRE-1α*, endoplasmic reticulum to nucleus signaling 1; *BiP*, heat shock protein family A (Hsp70) member 5; *PERK*, eukaryotic translation initiation factor 2-alpha kinase 3; *ATF-4*, activating transcription factor 4; and *CHOP*, DNA damage inducible transcript 3). All of these results clearly demonstrated the cytoprotective role of the DADLE peptide.

One year later, the same research group [21] investigated the effect of DADLE on hUCB-MSCs in another pathological condition, the hypoxia–reperfusion (H/R)-mimicked condition induced by cobalt chloride (CoCl_2_) treatment [100,101].

Data obtained confirmed the cytoprotective effect of DADLE even in this condition. To this end, DADLE significantly improved cell viability and alleviated ROS production and the activity of the mitochondrial complex 1 known to be associated with the production of ROS.

DADLE was also able to reduce apoptosis of hUCB-MSCs induced by the H/R-like stress through the upregulation of the anti-apoptotic gene *Bcl-2* and the downregulation of the pro-apoptotic gene *Bax*. In addition, DADLE was able to downregulate the levels of PERK, IRE-1*α*, ATF-6, and BiP, thus, modulating UPR protein levels. Moreover, in DADLE-treated hUCB-MSCs, the levels of secreted anti-inflammatory cytokines (IL-4, IL-10, and TGF-β) were upregulated, while a mitigation in pro-inflammatory cytokines (TNF-α; IFN-γ, interferon gamma; IL-6; and IL-1) secreted by hUCB-MSCs was observed when cells treated with DADLE were co-cultured with a murine macrophage cell line, RAW-264.7, under H/R-like conditions. Finally, the authors demonstrated that the cytoprotective effects exerted by DADLE were mediated by DOR since they were abrogated by the DOR-specific antagonist naltrindole [21].

## 3. Endogenous Opioids and Stem Cell Differentiation

The ability to differentiate is one of the most important properties of SCs. Here, we collect and summarize the results obtained from the studies demonstrating the involvement of endogenous or synthetic opioids in SC commitment and/or differentiation.

### 3.1. Neural Differentiation and Endogenous Opioids

Several studies suggested that endogenous opioids, through the three ORs (MOR, DOR, and KOR), could be involved in neurogenesis [102,103]. Here, we explore the literature about the role of endogenous opioids in SC dynamics and their ability to modulate neuronal differentiation (Table 2).

In 2006, Kim and colleagues [104] took into account that hormones, neurotransmitters, and growth factors can modulate cell proliferation and differentiation of self-renewing SCs and NPCs in several cell types via extracellular signal-regulated kinase (ERK) signaling [105,106]. Therefore, they firstly investigated whether the two endogenous ORs, MOR isoform 1 (MOR-1) and KOR isoform 1 (KOR-1), were expressed in mouse embryonic stem cells (ESCs) and then they attempted to dissect the underlying cell signaling pathways. By three independent approaches (qRT-PCR, immunoblot analysis of cell lysates, and immunofluorescence microscopy), these authors demonstrated that MOR-1 and KOR-1 were expressed in undifferentiated ESCs and in retinoic acid (RA)-induced ESC-derived NPs at different stages of development. Moreover, after 24 h of treatment of undifferentiated ESCs with the MOR-selective opioid agonist DAMGO or with the KOR-selective opioid agonist N-methyl-2-phenyl-N-[(5R,7S,8S)-7-(pyrrolidin-1-yl)-1-oxaspiro[4.5]dec-8-yl]acetamide (U69,593), ERK signaling was clearly activated.

In addition, they investigated whether ERK activation might influence ESC proliferation and differentiation. Undifferentiated ESCs (in the absence of opioids) can proliferate even when ERK signaling is blocked, indicating that the ERK/MAPK signaling pathway may not be involved in ESC self-renewal. On the other side, data suggested that DAMGO and U69,593 deviated ESCs from self-renewal and induced cells to differentiate. In RA-differentiated ESCs, opioid-induced signaling featured a biphasic ERK activation profile, as well as an opioid-induced ERK-independent inhibition of proliferation in these NPCs. Collectively, data suggested that opioids may have divergent effects on the self-renewal and differentiation processes of ESCs and that the latter only requires ERK activation. The authors indicated that the opioid modulation of ERK activity was an important regulator in ESC fate decisions by directing the cells to specific lineages [104].

A few years later, the same research group [107] studied the mechanisms that regulate the terminal differentiation of ESC-NPs. They first detected MOR and KOR immunoreactivity in NP-derived oligodendrocytes during all three stages of their in vitro maturation [108,109]. Then, they added DAMGO or U69,593 to the growth media of RA-induced mouse ESC-derived NPs and noticed a 50–60% reduction in neurogenesis or astrogenesis compared to controls [107]. By investigating the molecular mechanisms underlying these effects, the authors found that the opioid inhibition of NP-derived astrogenesis was driven via ERK signaling, while the p38 MAPK pathway was implicated in opioid attenuation of neurogenesis. In addition, MOR and KOR stimulated oligodendrogenesis from NP-derived oligodendrocyte progenitors via both ERK and p38 signaling pathways. These results indicate that MOR and KOR agonists may play a multifaceted modulatory role in NP terminal differentiation [107].

In 2006, Narita and collaborators [110] explored the role of ORs in neurogenesis using multipotent NSCs obtained from embryonic C3H mouse forebrains (MEB5); these cells are a multipotent SC line that can differentiate into neurons, astrocytes, and oligodendrocytes. Cells were treated with specific DOR, MOR, and KOR agonists, specifically (+)-4-[(alphaR)-alpha-((2S,5R)-4-allyl-2,5-dimethyl-1-piperazinyl)-3-methoxybenzyl]-N,N-diethylbenzamide] (SNC80), DAMGO, and [(-)-trans-(1S,2S)-U-50488 hydrochloride] (U50,488H), respectively. Only DOR activation promoted neural differentiation, as revealed by the significant increase in the levels of MAP2ab-like immunoreactivity and in the number of cell bodies, as well as the development of a complex dendritic tree with multiple secondary and tertiary dendrites. DOR activation, in the same model, also exerted a neuroprotective effect, as shown by a suppression of the apoptotic program induced by H_2_O_2_ treatment in cortical neuron/glia co-cultures [110].

To find candidates for cell therapy and to screen drugs for enkephalinergic disorders, such as autism, Hafizi and collaborators [111] used two in vitro SC models: unrestricted somatic stem cells (USSCs) and human bone marrow MSCs (BM-MSCs). Both SCs were differentiated into neuron-like cells and assessed for the expression of some enkephalinergic markers (Ikaros, CREBZF, and PENK). All markers were upregulated in USSC- and MSC-derived neurons, except for Ikaros in MSCs; moreover, in USSC-derived neurons, the expression of these markers was higher than in MSC-derived neurons. In addition, the analysis of PDYN expression in a neurogenic, differentiated state of USSCs revealed that this dynorphin-related marker was upregulated too, indicating USSCs to be potent neurogenic cells that express selected members of two opioid families (the enkephalins and the dynorphins) under the control of Ikaros activation [111].

In 2016, Trivedi and colleagues studied the neurogenesis related to morphin and thiol metabolites, redox status, and global DNA methylation levels [112]. They used NSC neurogenesis as an experimental model and exposed cells to morphine before evaluating parameters of interest. They first demonstrated that morphine promoted neurogenesis, increased apoptosis, and decreased total cell number during the later stages of differentiation. Thus, they showed that morphine treatment was associated with an increase in the glutathione/glutathione disulfide ratio and a decrease in the S-adenosylmethionine/S-adenosylhomocysteine ratio, then, a decrease in DNA methylation, indicating that morphine-regulated neurogenesis is associated with changes in redox state and epigenetic regulation [112]. 

Recently, the role of KOR agonists U50,488H and dynorphin-A (typical exogenous and endogenous agonists of KOR, respectively) in neurogenesis was further investigated [113]. Xu and collaborators showed that, in mouse hippocampal NSCs obtained from the hippocampi of 8-week-old animals, as well as from the hippocampus tissue of adult mice, the expression of KOR is significantly higher than that of MOR and DOR [113]. These authors then investigated the role of KOR agonists on proliferation, apoptosis, and differentiation of primary mouse NSCs. Data showed that the treatment of mouse NSCs with U50,488H or dynorphin-A did not influence cell proliferation and apoptosis [113]. On the contrary, both KOR agonists hindered adult neurogenesis by partially inhibiting neuronal differentiation of mouse NSCs. As a matter of fact, by monitoring the expression of nestin and Tuj1, two markers of NSCs and immature neurons, respectively, the authors found higher percentages of nestin-positive cells and lower percentages of Tuj1-positive cells, suggesting that both opioids partially inhibit neuronal differentiation. On the other hand, glia-specific and oligodendrocytes-specific differentiations were not affected. All these effects were blocked by nor-BNI, suggesting the pivotal role of KOR in neuronal differentiation [113].

To better understand the mechanism by which KOR regulates adult neurogenesis, the expression of neurogenesis-related genes was investigated. By modulating paired box 6/neurogenin 2/neuronal differentiation 1 (Pax6/Neurog2/NeuroD1) activities via upregulation of miR-7a expression, KOR agonists were found to hinder the neuronal differentiation of mouse NSCs and, hence, inhibit adult neurogenesis in the mouse hippocampus [113].

Overall, these results indicate that the opioid systems are differently involved in the regulation of neurogenesis; in particular, KOR is associated with a reduction in neural differentiation and astrogenesis [107,113], while MOR and DOR promote neural differentiation [106,110] respectively.

### 3.2. Hematopoietic and Vascular Stem Cell Differentiation and Endogenous Opioids

Many studies demonstrated the ability of SC to differentiate into hematopoietic and vascular lineages; in some of them, the involvement of endogenous opioids was addressed. These studies are summarized in this section and reported in Table 2.

As early as 1987, Skelly and colleagues [114] investigated the neuropeptide modulation on the hematopoietic differentiation in murine BM-MSCs. The authors observed that the simultaneous treatment of BM-MSCs with erythropoietin (in a suboptimal concentration) and with met- or leu-enkephalin, alpha- or beta-endorphin, or dynorphin enhanced the differentiation of these cells into erythroid colony-forming units (CFUs-e) [114]. However, it was observed that the hormone stimulation was necessary for the initiation of the differentiation process and that the peptides acted by modulating the formation of CFUs-e. Although the authors did not clarify the mechanism of action of the endorphins, these results documented the involvement of neuropeptides, particularly beta-endorphin, on the differentiation of mouse BM-MSCs into erythroid progenitors [114].

Many years later, murine BM progenitors were used to obtain dendritic cells (BM-derived DCs) and then employed to study the role of met-enkephalin in DC differentiation and, consequently, in the immunological response [115]. Indeed, DCs can be classified into myeloid dendritic cells (mDC), which preferentially elicit the type 1 T helper (Th1) response, and plasmacytoid dendritic cells (pDC), which promote a Th2 response. Liu and collaborators [115] showed that met-enkephalin, which promotes the expression of DOR and KOR in BM-derived DCs, induced them to polarize mainly towards the mDC subtype, rather than pDC, favoring the Th1 response. Furthermore, met-enkephalin upregulated the expressions of major histocompatibility complex (MHC) class II and key costimulatory molecules in BM-derived DCs and induced cells to produce higher levels of pro-inflammatory cytokines (active heterodimeric interleukin 12, IL-12p70; TNF-α) [115]. 

A few years later, it was observed that opioids and ORs were expressed in blood vessels in the later stage of rat embryos (16-embryonic-day-old) up to the adulthood [116,117]. The addition of opioid peptides was demonstrated to inhibit angiogenesis in a chick chorioallantoic membrane model [118] and DNA synthesis in rat vascular walls [106]. In the adult rat, the endogenous opioid system was shown to be active in hemodynamic and cardiovascular stress responses, such as hemorrhagic shock, sepsis, and trauma [119]. Moreover, the selective KOR agonist U50,488H exhibited beneficial effects on vascular injury after spinal cord trauma by improving vascular permeability and edema [120]. These findings suggest that the opioid system plays an important role in vascular functions, though its physiologic roles and molecular mechanisms remain largely unknown.

In 2011, Yamamizu and colleagues [121] investigated the role of opioids in vascular differentiation by using ESCs and two knockout mice models.

They obtained ESC-derived Flk1^+^ (fetal liver kinase 1/VEGF receptor-2^+^) vascular progenitors that expressed high levels of KOR but not MOR or DOR. The addition of KOR agonists to these cells inhibited endothelial cell (EC) differentiation and three-dimensional vascular formation. Since KOR agonists decreased the expression of Flk1 and Neuropilin1 (NRP1) in vascular progenitors, and the inhibitory effects of KOR were reversed by cAMP or PKA (protein kinase A) agonists, the authors concluded that the inhibitory effect of KOR agonists on vascular development could be due to an inhibition of cAMP/PKA signaling, followed by a decrease in Flk1 and NRP1 [122].

In addition, KOR-null or dynorphin-null mice showed a significant increase in vascular formation, together with an increase of Flk1 and NRP1 in their ECs, confirming the inhibitory role of KOR in vascular formation.

A few years later, Abdyazdani and colleagues investigated the effect of morphine on rat NSCs and on their ability to induce vasculogenesis [123]. Morphine strongly reduced rat NSC survival and clonogenicity, negatively affecting angiogenesis by the inhibition of the trans-differentiation of rat NSCs into vascular cells. All the effects induced by morphine treatment were attenuated in naloxone-treated rat NSCs [123].

### 3.3. Endogenous Opioid Dynorphin-B and Cardiac Differentiation in Stem Cells

The relationship between endogenous opioid and SC cardiac commitment was investigated, focusing mainly on the role of the dynorphin-B and of its coding gene *PDYN* (Table 2).

The first evidence that the opioid system plays a role in cardiac system dates back to 1989, when it was demonstrated that both DOR and KOR were expressed in the sarcolemma of myocardial cells [124]. Subsequently, many other observations led to the hypothesis that ORs are involved in the regulation of myocardial cells function. In fact, in rat cardiac myocytes, KOR and DOR stimulation increased cytosolic pH and myofilament responsiveness to Ca^2+^, affected phosphoinositide turnover [125], and influenced contractile dynamics in cardiomyocytes, as well as Ca^2+^ release from an intracellular pool in both cardiomyocytes and neurons [126,127]. Moreover, endorphins were reported to affect the contractility in isolated rat hearts [128,129], in cultured chick embryo heart cells [130,131], and in dog myocardia [132].

It is noteworthy that a large amount of PENK mRNA was detected in the rat heart tissue, in greater quantities than in any other tissues, including the brain, despite the cardiac content of the peptide preproenkephalin-A being significantly lower in the heart compared to the brain [133,134].

These findings induced researchers to even investigate the expression of *PDYN* gene and its related peptides in the myocardial cells. It was found that adult rat ventricular cardiomyocytes expressed the *PDYN* gene, and its bioactive end-product, dynorphin-B, was identified both intracellularly and in the culture medium [30].

Moreover, the presence of KOR was discovered in highly purified nuclei isolated from myocardial cells; in addition, when they were exposed to dynorphin-B, a significant increase in *PDYN* gene transcription was observed. These findings suggest the presence of a nuclear endorphinergic system as part of an intracrine loop [135].

All these studies were the premise for the subsequent studies on the PDYN system in SCs described below.

Ventura and collaborators detected both *PDYN* gene expression and intracellular and dynorphin-B protein secretion in ESCs [136]. Moreover, when ESCs were treated with dimethyl sulfoxide (DMSO) to promote cardiogenesis, an increase in *PDYN* gene expression and dynorphin-B synthesis and secretion was observed prior to the mRNA transcription of the cardiogenic lineage GATA binding protein 4 (*GATA-4*) and Nkx homeobox 5 (*Nkx-2.5*) [136]; these data indicate the chance for the intracrine and autocrine action of dynorphin-B to trigger the cardiogenic process [136]. Interestingly, dynorphin-B-conditioned medium was able to elicit *GATA-4* and *Nkx-2.5* gene transcription, even in the absence of DMSO, also enhancing the gene and protein expression of α-myosin heavy chain (α-MHC) and of the myosin light chain (MLC)-2V, two markers of terminal cardiac differentiation [136]. These findings were sustained by the possibility of the production of a cardiogenic SC line based upon lentiviral-mediated over-expression of the *PDYN* gene in mouse ESCs [137].

Evidence for a putative role of an endorphinergic system in cardiogenesis was also found in mouse GTR1-ESCs. In fact, KOR was found both on mouse GTR1-ESC plasma membranes [138] and nuclei [139], with a significant increase in the maximal binding capacity in nuclei isolated from GTR1-ESCs-derived cardiomyocytes compared with nuclei from undifferentiated cells [139]. At the same time, immunoreactive dynorphin-B was detected around the nucleus in undifferentiated ESCs and, highly enhanced, in GTR1-ESCs-derived cardiomyocytes [139]. Following these experiments, Ventura and colleagues found that the direct exposure to dynorphin-B of nuclei isolated from undifferentiated GTR1-ESCs was able to induce an increase in *GATA-4* and *Nkx-2.5* transcription rate but failed to affect the transcription of both myogenic differentiation 1 (*MyoD*) and *Neurog1* (involved in myogenesis and neurogenesis, respectively), indicating that the intracrine action of dynorphin-B maintains a high degree of selectivity [139]. Moreover, dynorphin-B was able to enhance the transcription of the *PDYN* gene in the nuclei isolated from undifferentiated mouse GTR1-ESCs, further highlighting the ability of this intracrine endorphinergic system to operate in a feed-forward manner [139]. The cardiogenesis primed by endogenous dynorphin-B in GTR1-ESCs was mediated by protein kinase C (PKC) signaling [138]; cardiac differentiation was associated with the enhancement of PKC-α, -β1, and -β2 expression and to their nuclear translocation from cytoplasm, while the enhanced expression of PKC-δ and -ε was mainly restricted to ESC nuclei [138,140]. To confirm this evidence, inhibition of the cell permeant PKC caused a complete abolition of cardiogenesis, and the exposure of GTR1-ESC nuclei to dynorphin-B caused an increase in nuclear PKC activity [139].

All these findings supported the evidence that dynorphin-B plays an important role in ESC cardiogenesis and that nuclear KOR and nuclear PKC signaling are coupled to SC cardiac commitment through the realization of an intracrine circuitry.

To better investigate cardiogenesis in SCs, Ventura and colleagues developed a combined molecule, hyaluronan mixed esters of butyric and retinoic acids (HBR), with the aim of making available a synthetic cardiac/vascular lineage-promoting agent [141]. In mouse ESCs, HBR induced the over-expression of *GATA-4* and *Nkx-2.5* genes, the increase in *PDYN* gene transcription, and the enhancement of the intracellular level of dynorphin-B [141]. These effects were already evident at the stage in which cells were grown as embryoid bodies in suspension and persisted in ESC-derived cardiomyocytes [141].

Experimental evidence of SC cardiogenic commitment associated with the role of the *PDYN* gene and dynorphin-B was also found in stem cells modulated by biophysical energies.

Magnetic fields (MFs) often modulate endogenous opioid peptide networks in several biological processes, such as the analgesic effects in in vivo animal models [142], the spontaneous electrical activity in the brain associated with opioids administration [143], or the central cholinergic activity in the rat mediated by MF [144].

To study the effects of MF on cardiogenesis, Ventura and colleagues drove an extremely low frequency MF (ELF-MF) of 50 Hz, 0.8 mTrsm, on adult rat myocardial cells and found that cardiomyocyte increased the PDYN mRNA level after MF exposure [145]. Moreover, when ELF-MFs were applied on mouse ESCs, an increase of *GATA-4* and *Nkx-2.5* expression gene was observed, leading to a strong increase in the number of spontaneously beating, ESC-derived cardiomyocytes. Interestingly, an enhancement in *PDYN* gene expression, as well as an increase in the intracellular levels of both dynorphin-B and of the dynorphin-B secretion in the culture medium, was found [146].

After these results, other experiments based on physical energies were performed on SCs; a radioelectric asymmetric conveyer (REAC) generating an extremely weak microwave emission (frequencies of 2.4 and 5.8 GHz) was used on mouse ESCs and human adipose-derived MSCs (ASCs). REAC modulated the transcription of stemness genes and increased the cells’ ability to differentiate into cardiac, neural, skeletal, and myogenic lineages. Moreover, REAC elicited an enhancement in GATA-4 and Nkx-2.5 expression in both mouse ESCs and human ASCs, which was still associated with an increase in PDYN gene transcription [147,148].

Consistent with these reports, Feridooni and colleagues confirmed the cardiogenic effect of dynorphin-B in mouse embryonic cardiac progenitor cells (CPCs), a reservoir of resident SCs [149]. In fact, dynorphin-B treatment induced in CPCs a remarkable increase in the number of CPC-derived cardiomyocytes.

Another research group recently elucidated the role of ORs and opioid peptides in the cardiomyogenesis of mouse ESCs [150]. They used a model of the spontaneous cardiomyogenic differentiation of mouse ESCs via the formation of embryoid bodies; both KOR and DOR significantly increased during ESC differentiation both in the nucleus and in the cytoplasmic membrane. Moreover, dynorphin-B inhibited the octamer-binding transcription factor 4 (Oct-4) mRNA transcription and increased the cardiomyocyte-specific *Nkx-2.5* gene expression, while dynorphin-A, met-enkephalin, and leu-enkephalin showed no significant effects on mouse ESC differentiation [150].

## 4. Conclusions

Overall, opioidergic systems encompass a wide-ranging variety of bioactive peptides, providing multi-layered control of major determinants in cell and SC biology. Compounding their biological complexity, opioid peptides were found to act as “one component–multiple target conductors”, which often led to the observation of opposite effects on the same outcome (i.e., proliferation or differentiation) depending on the specific SC target towards which activity was probed.

Nevertheless, deciphering the complexity of the informational cues associated with opioid peptide-mediated responses may hold promise for intriguing future developments. These future perspectives involve the potential for the timely and synergistic use of naturally occurring and synthetic opioids for the fine tuning of remarkable developments in regenerative medicine, including differentiation, proliferation, multicellular cross talk, inflammation, and tissue remodelling.

## Figures and Tables

**Table 1:** Effects of endogenous opioids on stem cell proliferation and stress response.

Opioids/Agonists	Pre-Treatment	Antagonists	Opioid Receptor	Cell Type	Biological Effects	Ref.
Met-enkephalin Morphine (10^−6^ M)		Naloxone (3 × 10^−6^ M)	DOR MOR	NPCs (from EGL of postnatal 5- and 6-day-old mice)	Morphine significantly reduced DNA content; this effect was attenuated by naloxone co-administration. Met-enkephalin did not alter DNA synthesis. Opioids did not affect cell viability.	[77]
Met-enkephalin (10^−6^ or 10^−5^ M)			MOR	hCB-CD34^+^ and hPB-CD34^+^ cells	hCB-CD34^+^ expressed MOR more than hPB-CD34^+^ cells. In treated hCB-CD34^+^ cells, phospho-MAPK was increased by 4.7- to 6.1-fold compared to the untreated cells; the increase of phospho-p38 was moderate. In hCB-CD34^+^, met-enkephalin did not reducethe apoptosis induced by irradiation.	[78]
Dynorphin-A[1–17] Dynorphin-A[2–17] U50,488 (10^−14^ to 10^−8^ M)		Nor-BNI (10^−6^ M)	KOR	NPCs (from 7- to 9-week-old human fetal brain tissue)	Dynorphin-A[1–17] and U50,488 stimulated cell proliferation and migration in a dose-dependent manner.	[81]
Morphine			MOR	NSCs	Theoretical hypothesis: since morphine reduces testosterone levels, increases DHT levels, andover-expresses *p53* gene, it might prevent NSC proliferation.	[82]
Morphine sulfate (10^−6^ to 1.3 × 10^−5^ M)		Naloxone	MOR	NPCs (from 14-day-oldmouse embryos)	Morphine decreased proliferation of NPCs and induced the caspase-3 activity in a dose-dependent manner. Morphine induced neuronal differentiation of NPCs.	[88]
Nociceptin			NOP	Mouse SSCs and spermatocytes	Nociceptin is an upstream Sertoli cell transcription factor that regulates SSC self-renewal and spermatocyte meiosis.	[90]
Morphine (10^−4^ M)		Naloxone (5 × 10^−5^ M)	MOR	Rat NSCs	Morphine decreased NSC growth and increased apoptosis. Morphine reduced the secretion of insulin and insulin-like growth factors and downregulated insulin receptor expression.	[89]
DADLE (10^−7^ M)	Serum deprivation	Naltrindole	DOR	hUCB-MSCs	DADLE increased anti-apoptotic Bcl-2, decreased pro-apoptotic Bax/Bad, decreased the activated caspase-3, upregulated PI3K subunit p110γ, and activated Akt. DADLE upregulated the release of anti-inflammatory cytokines (IL-4, IL-10, and TGF-β) and downregulated the secretion of pro-inflammatory cytokines (TNF-α, IL-6, and IL-1).	[93]
DADLE (10^−7^ M)	H_2_O_2_ (6 × 10^−4^ M)		DOR	hUCB-MSCs	DADLE increased cell viability, upregulated the anti-apoptotic protein Bcl-2,and suppressed the pro-apoptotic proteins Bax/Bad. DADLE reduced intracellular ROS levels and AP sites. DADLE downregulated UPR genes: *IRE-1α*, *BiP*, *PERK*, *ATF-4*, and *CHOP*.	[98]
DADLE (10^−7^ M)	H/R induced by CoCl_2_ (7.5 × 10^−4^ M)	Naltrindole	DOR	hUCB-MSCs	DADLE increased cell viability and reduced intracellular ROS levels. DADLE suppressed mitochondrial complex 1 activity. DADLE upregulated the anti-apoptotic gene *Bcl-2* while downregulating the pro-apoptotic gene *Bax* and UPR genes *PERK*, *IRE-1α*, *BiP*, *PERK*, and *ATF-6*. DADLE upregulated the release of anti-inflammatory cytokines (IL-4, IL-10, and TGF-β) and downregulated the secretion of pro-inflammatory cytokines (TNF-α, IL-6, IFN-γ, and IL-1β).	[21]

DOR, δ opioid receptor; MOR, μ opioid receptor; NPCs, neural precursor cells; EGL, external granular layer; hCB- and hPB-CD34+ cells, human CD34+ hematopoietic stem cells obtained from umbilical cord and peripheral blood, respectively; phospho-MAPK, phosphorylated form of mitogen-activated protein kinase; phospho-p38, phosphorylated form of p38 mitogen-activated protein kinase; U50,488, trans-3,4-dichloro-N-methyl-N[2-(1-pyrolidinyl)cyclohexyl] benzeneacetamide methanesulfonate; Nor-BNI, nor-binaltorphimine; KOR, κ opioid receptor; NSCs, neural stem cells; DHT, dihydrotestosterone; p53, tumor protein p53; NOP, nociceptin/orphanin FQ receptor; SSCs, spermatogonial stem cells; DADLE, [D-Ala2, D-Leu5]-enkephalin; hUCB-MSCs, human umbilical cord blood-derived mesenchymal stem cells; Bcl-2, B-cell lymphoma 2; Bax, Bcl-2-associated X protein; Bad, Bcl-2-associated death promoter; PI3K, phosphoinositide 3-kinase; Akt, protein kinase B; H_2_O_2_, hydrogen peroxide; ROS, reactive oxygen species; AP sites, apurinic/apyrimidinic sites; UPR, unfolded protein response; IRE-1α, inositol-requiring enzyme 1 alpha; Bip, binding immunoglobulin protein; PERK, protein kinase R-like endoplasmic reticulum kinase; ATF-4, activating transcription factor 4; CHOP, C/EBP homologous protein; H/R, hypoxia/reperfusion; CoCl_2_, cobalt chloride; ATF-6, activating transcription factor 6; IL-4, interleukin 4; IL-10, interleukin 10; TGF-β, transforming growth factor-beta; TNF-α, tumor necrosis factor-alpha; IL-6, interleukin 6; IFN-γ, interferon gamma; IL-1β, interleukin 1 beta.

**Table 2:** Effects of endogenous opioids on stem cell differentiation.

Opioids/Agonists	Pre-Treatment	Antagonists	Opioid Receptor	Cell Type	Biological Effects	Ref.
**Neural Differentiation**						
DAMGO U69,593 (10^−7^–10^−6^ M)	RA neuralinduction		KOR-1 MOR-1	ESCs (from mouse blastocyst) ESCs (from ICM of 3.5-day-old mouse)	MOR-1 and KOR-1 were expressed in undifferentiated ESCs and in RA-induced ESC-derived NPCs. Both opioids induced ESC neuronal differentiation activating ERK pathway.	[104]
DAMGO U69,593 (10^−6^ M)	RA neural induction		KOR MOR	ESCs (from mouse blastocyst)	Opioids reduced neurogenesis and astrogenesis in RA-induced ESC-NPCs through p38 MAPK and ERK pathways, respectively. Opioids stimulated oligodendrogenesis via both ERK and p38 signaling pathways.	[107]
DAMGO SNC80 U50,488H (10^−7^–3 × 10^−5^ M)			DOR KOR MOR	MEB5 (from 14.5-day-old mouse forebrains)	Only the DOR agonist SNC80 promoted neural differentiation.	[110]
	Neural induction			Human USSCs and BM-MSCs	Neural induction increased enkephalinergic markers (Ikaros, CREBZF, and PENK), especially in USSC-derived neuron-like cells. PDYN expression was enhanced in USSC-derived neuron-like cells.	[111]
Dynorphin-A U50,488H (10^−6^ M)	Neural induction with opioid/ agonist	Nor-BNI (10^−5^ M)	KOR	NSCs (from 8-week-old mouse hippocampus)	NSCs expressed high levels of KOR. Opioid treatment decreased neurogenesis by modulating Pax6/Neurog2/NeuroD1 activities via upregulation of miR-7a expression. Opioid treatment did not alter astrogenesis and oligodendrogenesis. Opioid treatment did not affect proliferation and apoptosis.	[113]
Morphine (10^−5^ M)	Neural induction with opioid			NSCs (from postnatal p0 mouse hippocampus)	Morphine promoted neurogenesis, increased apoptosis, and decreased total cell number during the later stages of differentiation. Morphine increased glutathione/glutathione disulfide ratio and decreased S-adenosylmethionine/S-adenosylhomocysteine ratio.	[112]
**Hematopoietic and Vascular Differentiation**						
Beta-endorphin (1 to 1000 ng/mL) Dynorphin (1 ng/mL) Leu-enkephalin Met-enkephalin (100 ng/mL)	EP (0.4 U/mL) induced erythropoiesis with opioid			Mouse BM progenitor cells	In the presence of EP, opioids enhanced BM progenitor differentiation into CFU-e.	[114]
TRK820 U50,488H (10^−5^ M)	Vascular induction		KOR	ESstA-ROSA (engineered mouse ESCs)	KOR agonists inhibited EC differentiation and 3D vascular formation in ESC-derived vascular progenitor cells. KOR agonists decreased the expression of Flk1 and NRP1 through inhibition of cAMP/PKA signaling in vascular progenitor cells.	[121]
Met-enkephalin (10^−14^ to 10^−8^ M)			KOR DOR	Mouse BM progenitor cells	Met-enk upregulated the expression of KOR and DOR in BM-derived DCs. Met-enk induced BM-derived DCs to differentiate mainly towards the mDC subtype. Met-enk increased the expression of MHC class II molecules and the release of pro-inflammatory cytokines (IL-12p70, TNF-α).	[115]
**Hematopoietic and Vascular Differentiation**						
Morphine (10^−4^ M)		Naloxone (10^−4^ M)		Rat NSCs	Morphine reduced survival and clonogenicity, negatively affecting tubulogenesis properties of NSCs by the inhibition of neuro-angiogenesis trans-differentiation.	[123]
**Cardiac Differentiation**						
Dynorphin-B (10^−9^ to 10^−6^ M)	DMSO 1%		KOR	Mouse ESCs	DMSO increased *PDYN* gene expression and dynorphin-B synthesis and secretion. Dynorphin-B elicited *GATA-4* and *Nkx-2.5* gene transcription and enhanced gene and protein expression of α-MHC and MLC-2V.	[136]
Dynorphin-B (10^−8^ to 10^−6^ M)	Cardiac induction		KOR	GTR1-ESCs (engineered mouse ESCs)	ESC plasma membranes and nuclei expressed KOR-specific opioid binding sites. ESC-derived cardiomyocytes showed an increase in dynorphin-B around the nucleus. Dynorphin-B induced an increase of *GATA-4*, *Nkx-2.5*, and *PDYN* gene expressions and promoted cardiogenesis by PKC signaling.	[138,139]
	HBR cardiac induction (0.75 mg/mL)			GTR1-ESCs (engineered mouse ESCs)	HBR-induced ESC-derived cardiomyocytes enhanced *GATA-4*, *Nkx-2.5*, and *PDYN* gene transcriptions and the intracellular level of dynorphin-B.	[141]
	ELF-MF exposition during cardiac induction (50 Hz, 0.8 m Trms)			GTR1-ESCs (engineered mouse ESCs)	ELF-MF spontaneously induced cardiogenesis, upregulating GATA-4, Nkx-2.5, and PDYN gene expression and enhancing intracellular levels and secretion of dynorphin-B.	[146]
**Cardiac Differentiation**						
	REAC exposition during cardiac induction (MF of 2.4 and 5.5 GHz)			Mouse ESCs and human ASCs	Both SCs committed to cardiac lineage and exposed to REAC increased the expression of *GATA-4*, *Nkx-2.5*, and *PDYN* gene.	[147,148]
Dynorphin-B (10^−7^ M)	Cardiac induction			CPCs (from 11.5-day-oldembryonic mouseventricles)	Dynorphin B promoted CPC differentiation into cardiomyocytes.	[149]
Dynorphin-A Dynorphin-B Met-enkephalins Leu-enkephalins (10^−5^ M)	Cardiac induction		DOR KOR	Mouse ESCs	Both DOR and KOR increased during ESC differentiation. Dynorphin-B inhibited *Oct-4* and increased *Nkx-2.5* gene expression. Dynorphin-A, met-enkephalins, and leu-enkephalinsdid not affect ESC differentiation.	[150]

DAMGO, [D-Ala^2^,MePhe^4^,Glyol^5^]-enkephalin; U69,593, N-methyl-2-phenyl-N-[(5R,7S,8S)-7-(pyrrolidin-1-yl)-1-oxaspiro[4.5]dec-8-yl]acetamide; RA, retinoic acid; KOR-1, κ opioid receptor isoform 1; MOR-1, μ opioid receptor isoform 1; ESCs, embryonic stem cells; ICM, inner cell mass; NPCs, neural progenitor cells; ERK, extracellular signal-regulated kinase; p38 MAPK, p38 mitogen-activated protein kinase; SNC80, [(+)-4-[(alphaR)-alpha-((2S,5R)-4-allyl-2,5-dimethyl-1-piperazinyl)-3-methoxybenzyl]-N,N-diethylbenzamide]; U50,488H, (–)-trans-(1S,2S)-U-50488 hydrochloride; Nor-BNI, nor-binaltorphimine; DOR, δ opioid receptor; MEB5, multipotent neural stem cells; USSCs, unrestricted somatic stem cells; BM-MSCs, bone marrow mesenchymal stem cells; Ikaros, IKAROS family zinc finger 1; CREBZF, CREB/ATF bZIP transcription factor; PENK, proenkephalin; PDYN, prodynorphin; NSCs, neural stem cells; Pax6, paired box 6; Neurog2, neurogenin 2; NeuroD1, neuronal differentiation 1; leu-enkephalin, leucine-enkephalin; met-enkephalin, methionine-enkephalin; EP, erythropoietin; CFU-e, colony-forming unit-erythroid; TRK820, 17-cyclopropylmethyl-3,14β-dihydroxy-4,5α-epoxy-6β-[N-methyl-trans-3-(3-furyl) acrylamido]morphinan hydrochloride; EC, endothelial cell; Flk1, fetal liver kinase 1/VEGF receptor 2; NRP1, neuropilin 1; cAMP, cyclic adenosine monophosphate; PKA, protein kinase A; DCs, dendritic cells; mDCs, myeloid dendritic cells; MHC, major histocompatibility complex; TNF-α, tumor necrosis factor alpha; IL-12p70, active heteodimer of interleukin 12. p53, tumor protein p53; DMSO, dimethyl sulfoxide; GATA-4, GATA binding protein 4; Nkx-2.5, Nkx homeobox 5; α-MHC, α-myosin heavy chain; MLC-2V, myosin light chain; PKC, protein kinase C; HBR, hyaluronan mixed esters of butyric and retinoic acids; ELF-MF, extremely low frequency magnetic fields; REAC, radio electric asymmetric conveyer; ASCs, adipose-derived mesenchymal stem cells; SCs, stem cells; CPCs, cardiac progenitor cells; Oct-4, octamer-binding transcription factor 4.

## Data Availability

Data are available upon request from S.C., e-mail: silvia.canaider@unibo.it.
